# Detection of Squamous Cell Carcinoma Foci in a Patient with Dystrophic Epidermolysis Bullosa in ^18^F-FDG PET/CT

**DOI:** 10.4274/mirt.galenos.2018.96658

**Published:** 2019-06-24

**Authors:** Esra Arslan, Tevfik Fikret Çermik, Ayşe Esra Koku Aksu, Mehmet Salih Gürel, Cem Leblebici

**Affiliations:** 1 University of Health Sciences, İstanbul Training and Research Hospital, Clinic of Nuclear Medicine, İstanbul, Turkey; 2 University of Health Sciences, İstanbul Training and Research Hospital, Clinic of Dermatology, İstanbul, Turkey; 3 University of Health Sciences, İstanbul Training and Research Hospital, Clinic of Pathology, İstanbul, Turkey

**Keywords:** 18F-FDG PET/CT, dystrophic epidermolysis bullosa, squamous cell carcinoma

## Abstract

Dystrophic epidermolysis bullosa (DEB) is a rare, inherited skin fragility disorder characterized by blister formation in the sublamina densa. DEB is associated with aggressive squamous cell carcinoma (SCC) that has increased risk of metastases and poor prognosis. A 41-year-old woman with DEB underwent ^18^F-fluoro-2-deoxy-glucose positron emission tomography/computed tomography (^18^F-FDG PET/BT). PET/CT showed increased ^18^F-FDG uptakes in multifocal cutaneous lesions in both lower extremities. The patient was diagnosed with SCC via skin biopsy from the left lateral lower thigh. Ten months later, PET/CT showed increased FDG uptakes in the primary tumor area as well as the left inguinal and left supraclavicular lymph node regions. ^18^F-FDG PET/CT seems to be useful for re-staging and planning appropriate therapeutic strategy in DEB-patients with SCC.

## Abstract (tr)

Distrofik epidermolizis bülloza (DEB), sublamina densada veziküler ve yaygın bül oluşumu ile karakterize nadir görülen kalıtsal bir deri frajilite bozukluğudur. DEB bilhassa artmış metastaz riski ve kötü prognostik özellikler gösteren agresif skuamöz hücreli karsinom (SHK) oluşumu ile ilişkilendirilmiştir. DEB tanısı konulan 41 yaşındaki kadın olgu ^18^F-floro-2-deoksi-glukoz pozitron emisyon tomografisi/bilgisayarlı tomografi (^18^F-FDG PET/BT) taraması ile deri malignitesi açısından incelenmiştir. PET/BT taraması ile her iki alt ekstremitede multifokal kutanöz lezyonlarda artmış ^18^F-FDG tutulumu gösterilmiş ve sol lateral alt uyluk bölge derisinden elde edilen biyopsi materyali ile SHK tanısı konulmuştur. On ay sonra yapılan ikinci PET/BT taraması sonucunda primer tümör alanı, sol inguinal ve sol supraklaviküler lenf nodu alanında artmış ^18^F-FDG tutulumu gösterilmiştir. SHK’li DEB hastalarında ^18^F-FDG PET/BT yönteminin özellikle uygun terapötik stratejinin belirlenmesi adına hastalığın yönetimi ve yeniden evrelendirilmesinde faydalı olacağı kanaatindeyiz.

## Figures and Tables

**Figure 1:**
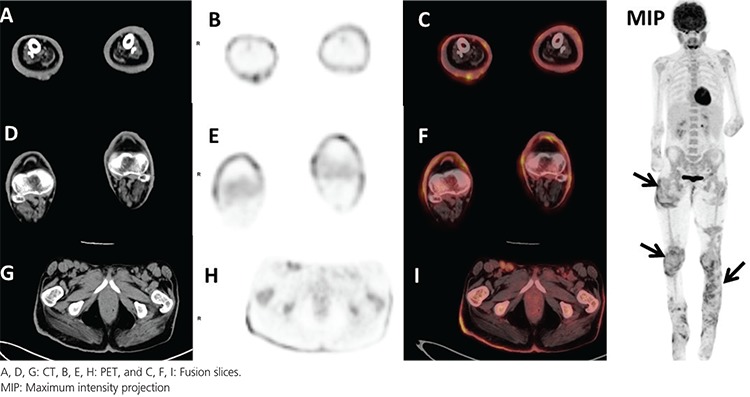
Epidermolysis bullosa (EB) is a clinically heterogeneous group of inherited blistering disorders characterized by increased skin fragility, while the dystrophic variant of EB (DEB) is a clinically more severe subtype of EB (1,2). Patients with DEB are at high risk of developing squamous cell carcinoma (SCC), which particularly arise from areas of poorly healing wounds, and lead to metastasis and death (3). A 41-year-old female patient with DEB underwent ^18^F-fluoro-2-deoxy-glucose positron emission tomography/computed tomography (^18^F-FDG PET/CT) in the follow-up period. PET/CT showed increased metabolic activity in multifocal cutaneous lesions in both lower extremities. The incisional biopsy performed from the skin of the left lateral lower thigh where one of the increased ^18^F-FDG uptakes was observed revealed invasive SCC (black arrows). There was no other increased pathologic metabolic activity in any part of the skin, lymph nodes or organs

**Figure 2:**
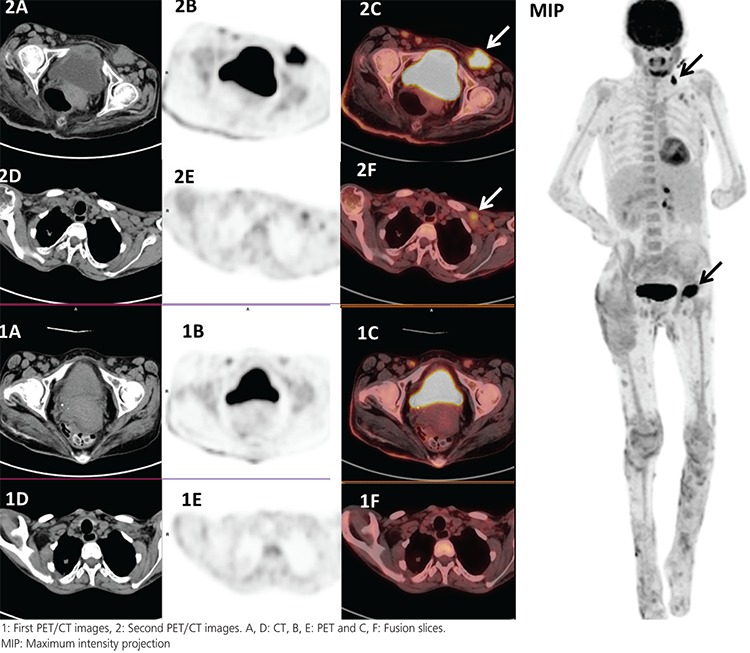
Ten months later, a second PET/CT scan was performed for re-staging purposes due to clinical detection of suspicious inguinal lymph nodes on physical examination. The second PET/CT showed a lymph node with increased FDG uptake in the left inguinal region (SUV_max_: 12.9) as well as additional unexpected lymph nodes in the left supraclavicular region (SUV_max_: 11.0) consistent with local and distant nodal metastasis (black arrows). Due to the multifocal or multiclonal onset of SCC, it is difficult to identify nodal and visceral spread of the tumor (4). Despite the high sensitivity of CT and PET/CT to detect subclinical nodal spread, false-positive results are still common. (5) By Jennings and Schmults ^18^F-FDG PET is reported to be beneficial to differentiate disease involvement and areas of necrosis and fibrosis. Cho et al. (6) have examined 12 SCC patients (nine cases with highrisk SCC) by ^18^F-FDG/PET. The authors have identified lymph node metastases in three cases (25.0%), distant organ involvement in one case (8.3%) and primary lesions in nine cases (83.3%). Mahajan et al. (7) reported that ^18^F-FDG PET/CT achieved overall sensitivity and accuracy of 100% and 92%, respectively, in 13 patients with primary SCC. It was emphasized that ^18^F-FDG detected four previously unknown secondary lesions and changed management schedule in three of these. Supportively, Mackie and Avram (8) evaluated a 34-year-old woman with EB with soft tissue thickening in the left foot showing an increased ^18^F-FDG uptake, which was confirmed histopathologically as SCC. In conclusion, ^18^F-FDG PET/CT seems to be useful in re-staging and management of follow-up to plan appropriate therapeutic strategy in DEB patients with SCC
